# The genome sequence of an ichneumon wasp, *Ophion slaviceki *(Kriechbaumer, 1892)

**DOI:** 10.12688/wellcomeopenres.19996.1

**Published:** 2023-09-08

**Authors:** Gavin R. Broad, Natural History Museum Genome Acquisition Lab, Darwin Tree of Life Barcoding collective, Wellcome Sanger Institute Scientific Operations: DNA Pipelines collective, Tree of Life Core Informatics collective, Darwin Tree of Life Consortium

**Affiliations:** 1 Natural History Museum, London, England, UK

**Keywords:** Ophion slaviceki, an ichneumon wasp, genome sequence, chromosomal, Hymenoptera

## Abstract

We present a genome assembly from an individual female *Ophion slaviceki* (an ichneumon wasp; Arthropoda; Insecta; Hymenoptera; Ichneumonidae). The genome sequence is 654.2 megabases in span. Most of the assembly is scaffolded into 13 chromosomal pseudomolecules. The mitochondrial genome has also been assembled and is 16.19 kilobases in length. Gene annotation of this assembly on Ensembl identified 19,399 protein coding genes.

## Species taxonomy

Eukaryota; Metazoa; Eumetazoa; Bilateria; Protostomia; Ecdysozoa; Panarthropoda; Arthropoda; Mandibulata; Pancrustacea; Hexapoda; Insecta; Dicondylia; Pterygota; Neoptera; Endopterygota; Hymenoptera; Apocrita; Ichneumonoidea; Ichneumonidae; Ophioninae; Ophion group; *Ophion*; *Ophion slaviceki* (Kriechbaumer, 1892) (NCBI:txid2979155).

## Background

*Ophion slaviceki* is a widely distributed and often common ichneumonid (Darwin Wasp), a parasitoid wasp of the cosmopolitan subfamily Ophioninae. Like most ophionines, *Ophion slaviceki* is primarily nocturnal, seeking night-active caterpillars. It possesses numerous features associated with nocturnal behaviour, such as a uniformly pale reddish colour, long antennae, and very large compound eyes and ocelli ( Gauld & Huddleston, 1976).

*Ophion slaviceki* is active almost exclusively in August and September, and is a parasitoid of *Agrotis* noctuid moths, particularly Heart and Dart ( *Agrotis exclamationis* (Linnaeus, 1758)) and Turnip Moth ( *Agrotis segetum* (Denis & Schiffermüller, 1775)) ( Broad *et al.*, 2015). The host larvae are well-grown at this time of year, emerging at night to feed on low vegetation. The *Agrotis* caterpillars feed periodically throughout winter and then form a pupation retreat in the soil in the spring. At this point, the *O. slaviceki* larva completes its feeding and emerges from the host to spin a densely woven, dark brown silken cocoon, from which the adult emerges later that year. As with its hosts, *O.slaviceki* seems to be very widespread across Britain and Ireland but distribution data are sparse ( Broad *et al.*, 2015). Adult *O. slaviceki* are separable from other similar *Ophion* species by a combination of a long trochantellus on the hind leg, mandibles with a simple, acutely angled gap between the teeth, and the fore wing venation, with a combination of a sinuous vein *RS* and a very short ramulus ( Brock, 1982; Johansson & Cederberg, 2019).

In Britain, *O*. *slaviceki* is one of 31 *Ophion* species found so far, with more to be discovered. When the checklist of British and Irish Ichneumonidae was published in 2016 ( Broad, 2016), only 16 species in the genus were recognised. Long known to be a taxonomically difficult genus, the use of DNA barcode data helped unravel various species complexes. Johansson and Cederberg (2019) overhauled the taxonomy of Swedish *Ophion*, with many of the newly described species present in the UK too. *Ophion slaviceki* has been called *Ophion luteus* (Linnaeus, 1758) in most publications, but the actual *O. luteus* is a smaller species which flies in early summer ( Johansson & Cederberg, 2019).

The genome of *O. slaviceki* is the first chromosomal level genome for the subfamily Ophioninae, and one of a small number for the ophioniformes group of subfamilies ( Wahl, 1991), a monophyletic group which is noteworthy for being koinobiont parasitoids (i.e., they allow their hosts to continue development following oviposition) and for repeated adoptions of viruses which have allowed these wasps to overcome host immune responses ( Legeai *et al.*, 2020). Comparative genomics should illuminate some of the adaptations which have allowed these wasps to exploit host insects in such intricate ways.

## Genome sequence report

**Figure 1.**
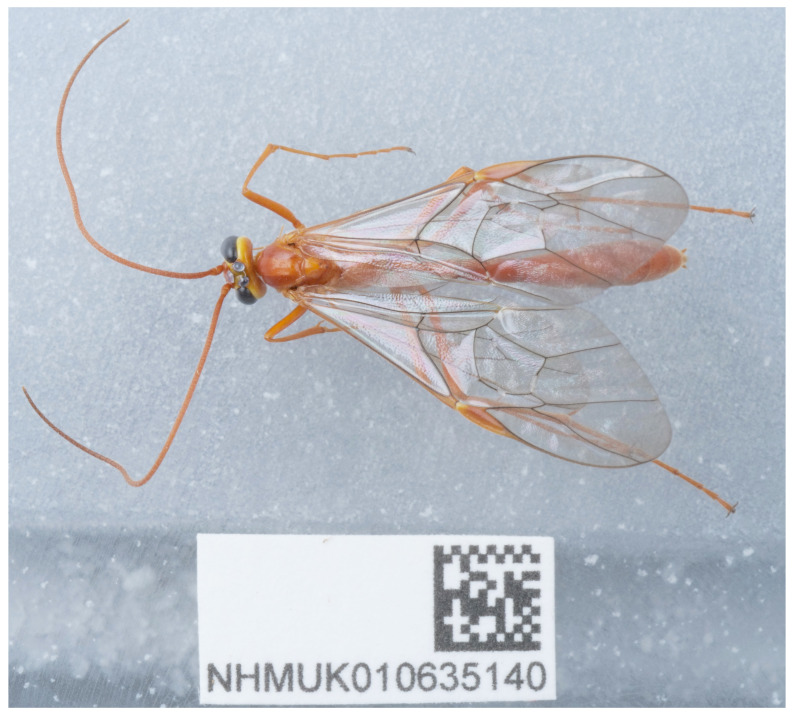
Photograph of the *Ophion slaviceki* (iyOphLute1) specimen used for genome sequencing.

The genome was sequenced from one female *Ophion slaviceki* ( Figure 1) collected from Tonbridge, England, UK (50.25, 1.64). A total of 35-fold coverage in Pacific Biosciences single-molecule HiFi long reads and 56-fold coverage in 10X Genomics read clouds were generated. Primary assembly contigs were scaffolded with chromosome conformation Hi-C data. Manual assembly curation corrected 1,749 missing joins or mis-joins and removed 2 haplotypic duplications, reducing the scaffold number by 63.81%, and increasing the scaffold N50 by 23.11%.

**Figure 2.**
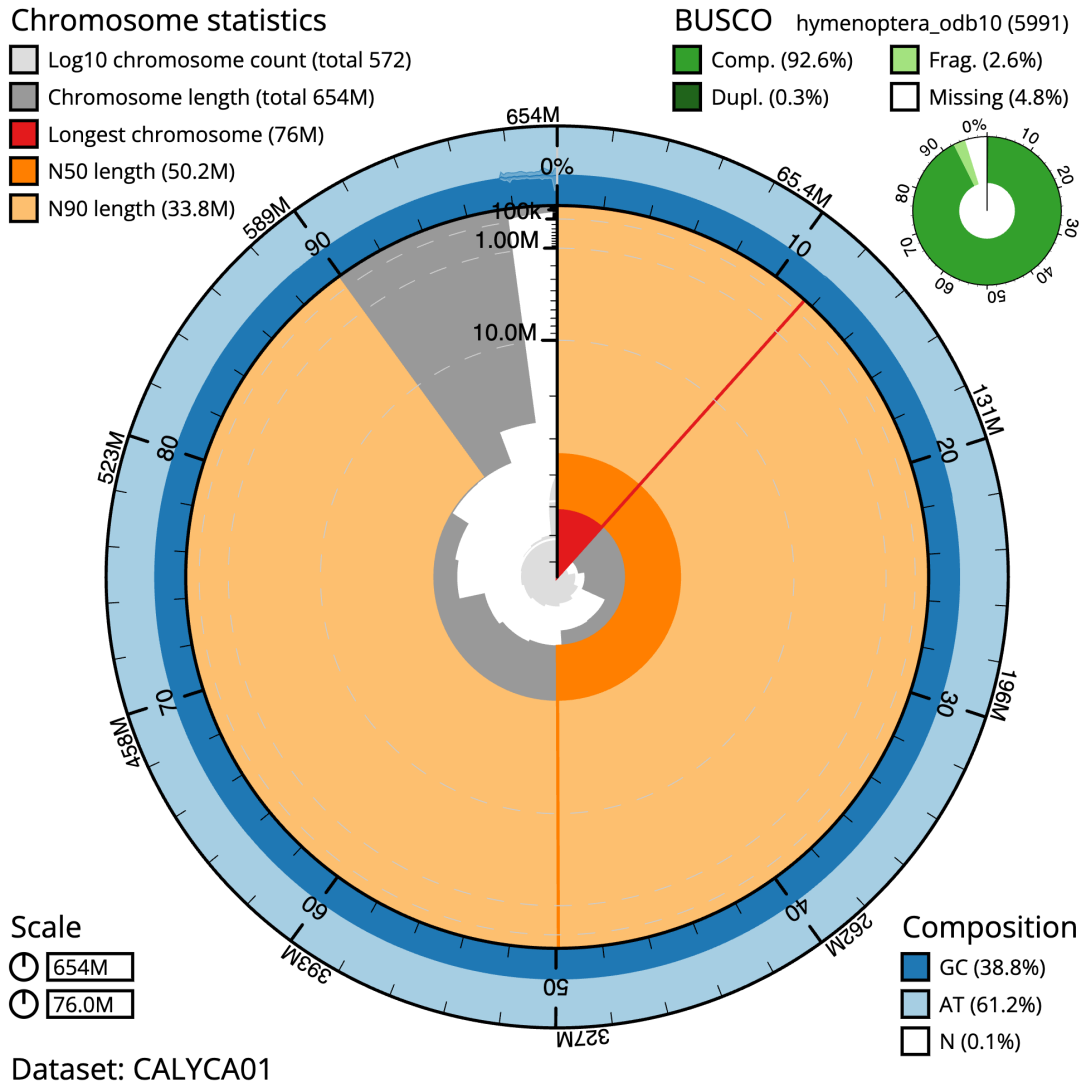
Genome assembly of *Ophion slaviceki*, iyOphLute1.1: metrics. The BlobToolKit Snailplot shows N50 metrics and BUSCO gene completeness. The main plot is divided into 1,000 size-ordered bins around the circumference with each bin representing 0.1% of the 654,238,417 bp assembly. The distribution of scaffold lengths is shown in dark grey with the plot radius scaled to the longest scaffold present in the assembly (75,994,433 bp, shown in red). Orange and pale-orange arcs show the N50 and N90 scaffold lengths (50,234,018 and 33,763,864 bp), respectively. The pale grey spiral shows the cumulative scaffold count on a log scale with white scale lines showing successive orders of magnitude. The blue and pale-blue area around the outside of the plot shows the distribution of GC, AT and N percentages in the same bins as the inner plot. A summary of complete, fragmented, duplicated and missing BUSCO genes in the hymenoptera_odb10 set is shown in the top right. An interactive version of this figure is available at https://blobtoolkit.genomehubs.org/view/iyOphLute1.1/dataset/CALYCA01/snail.

The final assembly has a total length of 654.2 Mb in 571 sequence scaffolds with a scaffold N50 of 50.2 Mb ( Table 1). Most (97.75%) of the assembly sequence was assigned to 13 chromosomal-level scaffolds. Chromosome-scale scaffolds confirmed by the Hi-C data are named in order of size ( Figure 2– Figure 5; Table 2). While not fully phased, the assembly deposited is of one haplotype. Contigs corresponding to the second haplotype have also been deposited. The mitochondrial genome was also assembled and can be found as a contig within the multifasta file of the genome submission.

**Table 1.** Genome data for *Ophion slaviceki*, iyOphLute1.1.

Project accession data		
Assembly identifier	iyOphLute1.1	
Species	*Ophion slaviceki*	
Specimen	iyOphLute1	
NCBI taxonomy ID	2979155	
BioProject	PRJEB53249	
BioSample ID	SAMEA8534284	
Isolate information	iyOphLute1, female: abdomen (DNA sequencing), head and thorax (Hi-C sequencing)	
Assembly metrics *		*Benchmark*
Consensus quality (QV)	50.5	*≥50*
*k*-mer completeness	99.97%	*≥ 95%*
BUSCO **	C:92.6%[S:92.2%,D:0.3%],F:2.6%,M: 4.8%,n:5,991	*C ≥ 95%*
Percentage of assembly mapped to chromosomes	97.75%	*≥ 95%*
Sex chromosomes	-	*localised homologous pairs*
Organelles	Mitochondrial genome assembled	*complete single alleles*
Raw data accessions		
PacificBiosciences SEQUEL II	ERR9836428, ERR9836429	
10X Genomics Illumina	ERR9820275, ERR9820276, ERR9820273, ERR9820274	
Hi-C Illumina	ERR9820277	
Genome assembly		
Assembly accession	GCA_944452715.1	
*Accession of alternate haplotype*	GCA_944452655.1	
Span (Mb)	654.2	
Number of contigs	2,836	
Contig N50 length (Mb)	0.5	
Number of scaffolds	571	
Scaffold N50 length (Mb)	50.2	
Longest scaffold (Mb)	76.0	
Genome annotation		
Number of protein-coding genes	19,399	
Number of gene transcripts	19,543	

* Assembly metric benchmarks are adapted from column VGP-2020 of “Table 1: Proposed standards and metrics for defining genome assembly quality” from ( Rhie *et al.*, 2021).

** BUSCO scores based on the hymenoptera_odb10 BUSCO set using v5.3.2. C = complete [S = single copy, D = duplicated], F = fragmented, M = missing, n = number of orthologues in comparison. A full set of BUSCO scores is available at https://blobtoolkit.genomehubs.org/view/iyOphLute1.1/dataset/CALYCA01/busco.

**Figure 3.**
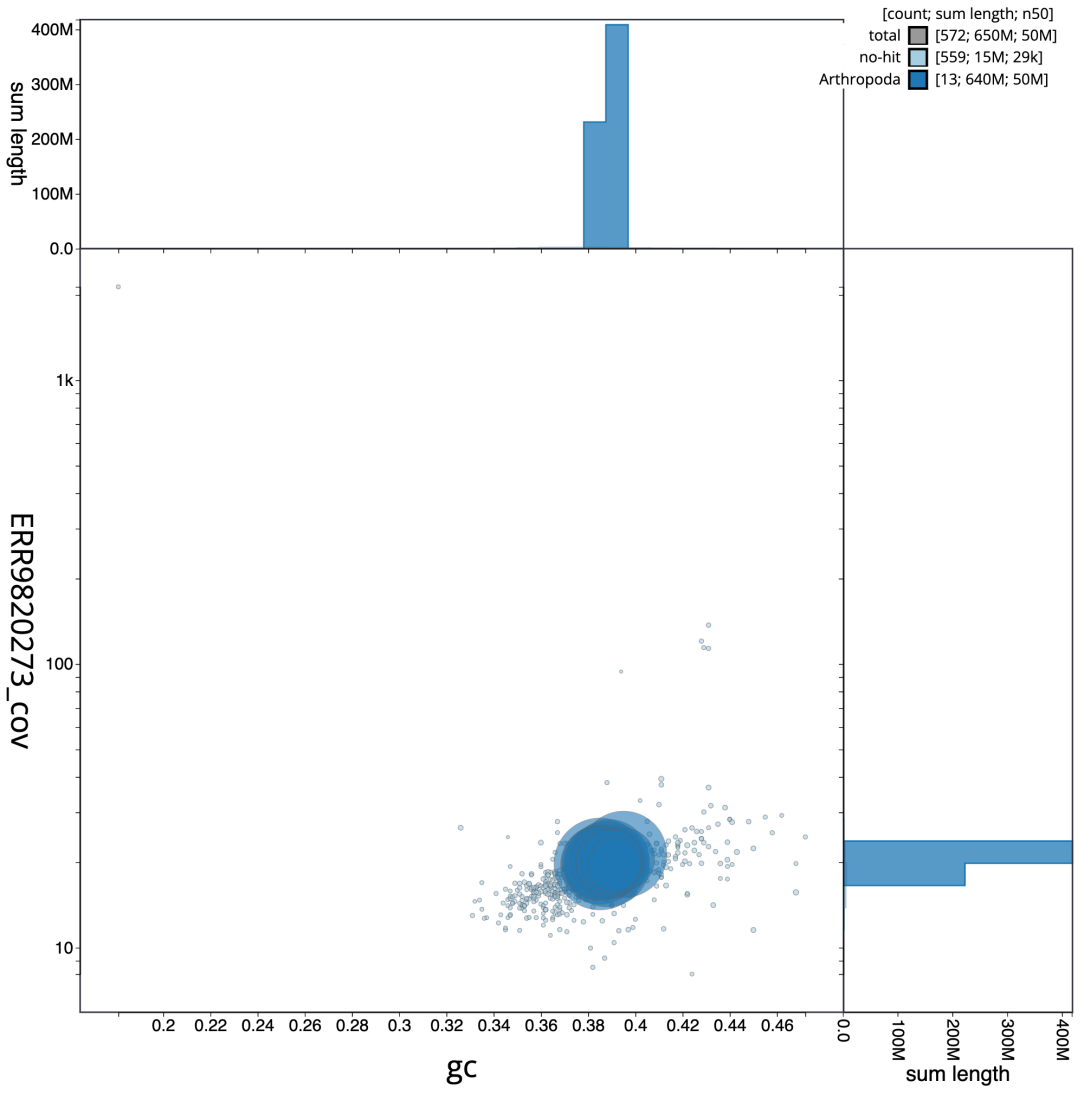
Genome assembly of *Ophion slaviceki*, iyOphLute1.1: BlobToolKit GC-coverage plot. Scaffolds are coloured by phylum. Circles are sized in proportion to scaffold length. Histograms show the distribution of scaffold length sum along each axis. An interactive version of this figure is available at https://blobtoolkit.genomehubs.org/view/iyOphLute1.1/dataset/CALYCA01/blob.

**Figure 4.**
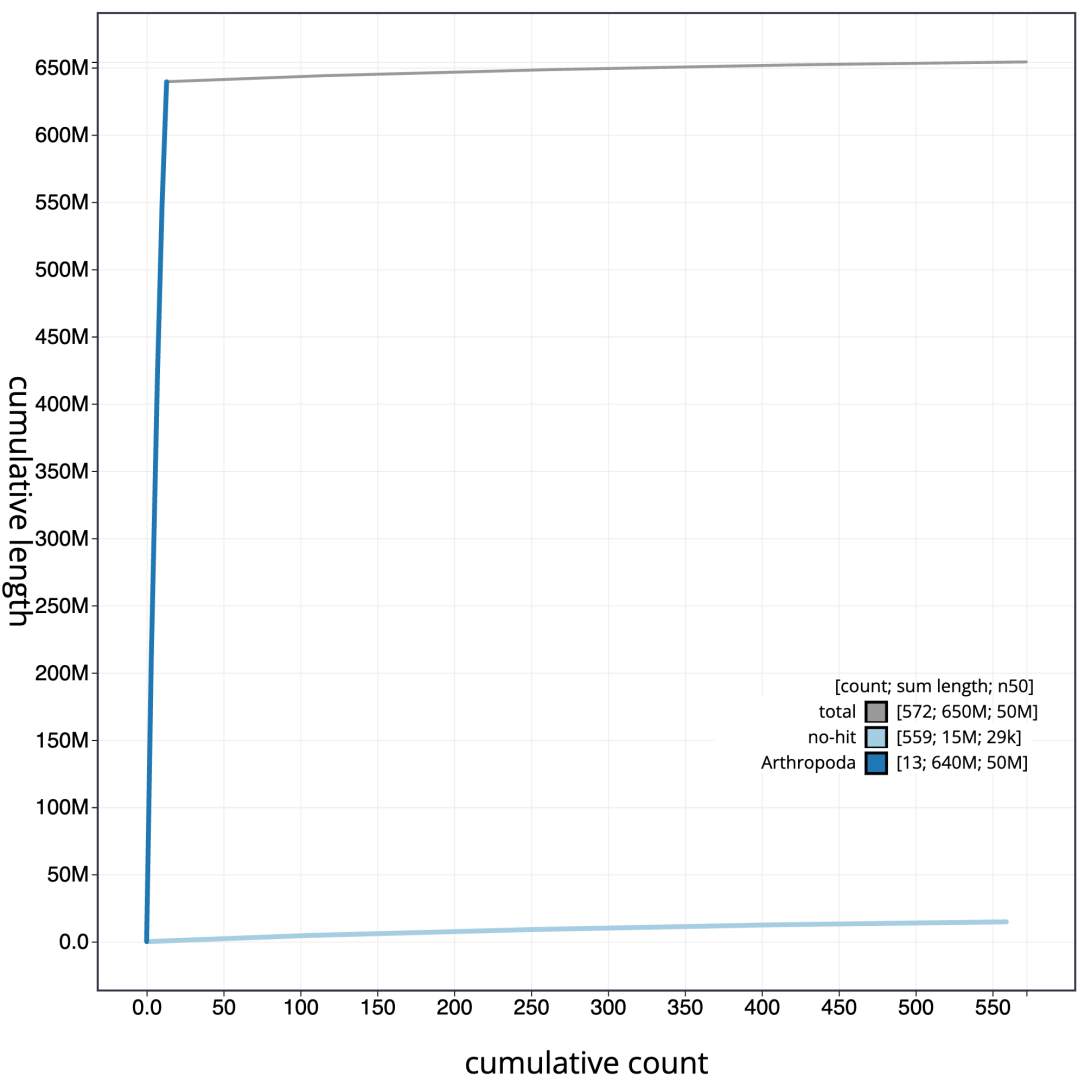
Genome assembly of *Ophion slaviceki*, iyOphLute1.1: BlobToolKit cumulative sequence plot. The grey line shows cumulative length for all scaffolds. Coloured lines show cumulative lengths of scaffolds assigned to each phylum using the buscogenes taxrule. An interactive version of this figure is available at https://blobtoolkit.genomehubs.org/view/iyOphLute1.1/dataset/CALYCA01/cumulative.

**Figure 5.**
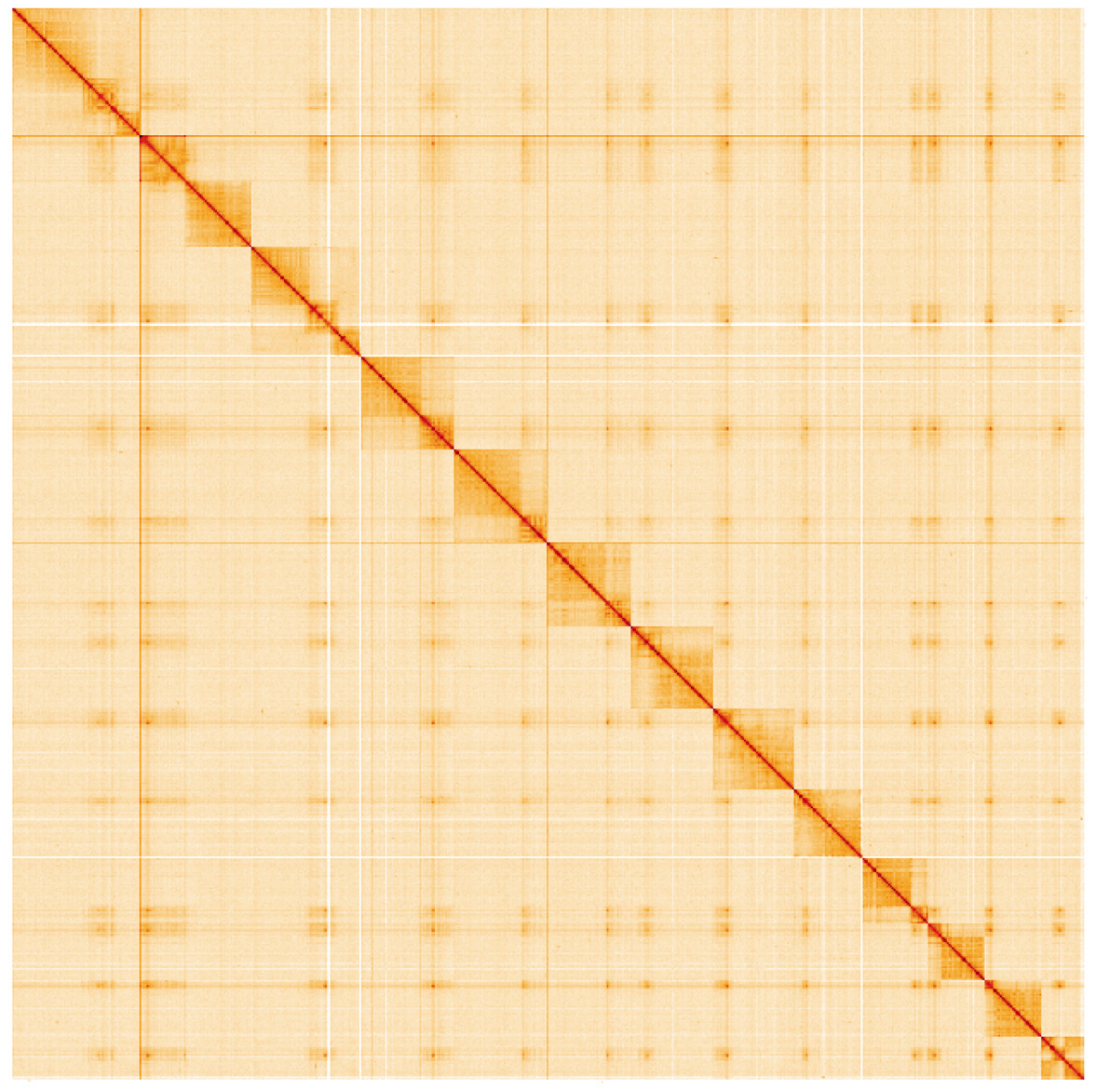
Genome assembly of *Ophion slaviceki*, iyOphLute1.1: Hi-C contact map of the iyOphLute1.1 assembly, visualised using HiGlass. Chromosomes are shown in order of size from left to right and top to bottom. An interactive version of this figure may be viewed at https://genome-note-higlass.tol.sanger.ac.uk/l/?d=Xdr6JEDrS4uD2QhUm0QmeQ.

**Table 2.** Chromosomal pseudomolecules in the genome assembly of *Ophion slaviceki*, iyOphLute1.

INSDC accession	Chromosome	Length (Mb)	GC%
OX101783.1	1	75.99	38.5
OX101784.1	2	67.16	39.5
OX101785.1	3	64.84	39.0
OX101786.1	4	55.58	39.0
OX101787.1	5	55.28	38.5
OX101788.1	6	50.23	38.5
OX101789.1	7	49.52	38.5
OX101790.1	8	48.28	39.0
OX101791.1	9	40.29	39.0
OX101792.1	10	39.27	39.0
OX101793.1	11	33.88	39.5
OX101794.1	12	33.76	39.0
OX101795.1	13	25.43	39.0
OX101796.1	MT	0.02	18.0

The estimated Quality Value (QV) of the final assembly is 50.5 with *k*-mer completeness of 99.97%, and the assembly has a BUSCO v5.3.2 completeness of 92.6% (single =92.2%, duplicated = 0.3%), using the hymenoptera_odb10 reference set ( *n* = 5,991).

Metadata for specimens, spectra estimates, sequencing runs, contaminants and pre-curation assembly statistics can be found at https://tolqc.cog.sanger.ac.uk/darwin/insects/Ophion_slaviceki/.

## Genome annotation report

The *Ophion slaviceki* genome assembly (GCA_944452715.1) was annotated using the Ensembl rapid annotation pipeline ( Table 1; https://rapid.ensembl.org/Ophion_slaviceki_GCA_944452715.1/Info/Index). The resulting annotation includes 19,543 transcribed mRNAs from 19,543 protein-coding genes.

## Methods

### Sample acquisition and nucleic acid extraction

A female *Ophion slaviceki* (specimen IDNHMUK010635140, ToLIDiyOphLute1) was collected from a garden in Tonbridge, Kent (latitude50.25, longitude 1.64)on 2020-08-20 using a light trap. The specimen was collected and identified by Gavin Broad (Natural History Museum) and snap-frozen in liquid nitrogen.

DNA was extracted at the Tree of Life laboratory, Wellcome Sanger Institute (WSI). The iyOphLute1 sample was weighed and dissected on dry ice with head and thorax tissue set aside for Hi-C sequencing. Tissue from the abdomen was disrupted using a Nippi Powermasher fitted with a BioMasher pestle. High molecular weight (HMW) DNA was extracted using the Qiagen MagAttract HMW DNA extraction kit. Low molecular weight DNA was removed from a 20 ngaliquot of extracted DNA using the 0.8X AMpure XP purification kit prior to 10X Chromium sequencing; a minimum of 50 ng DNA was submitted for 10X sequencing. HMW DNA was sheared into an average fragment size of 12–20 kb in a Megaruptor 3 system with speed setting 30. Sheared DNA was purified by solid-phase reversible immobilisation using AMPure PB beads with a 1.8X ratio of beads to sample to remove the shorter fragments and concentrate the DNA sample. The concentration of the sheared and purified DNA was assessed using a Nanodrop spectrophotometer and Qubit Fluorometer and Qubit dsDNA High Sensitivity Assay kit. Fragment size distribution was evaluated by running the sample on the FemtoPulse system.

### Sequencing

Pacific Biosciences HiFi circular consensus and 10X Genomics read cloud DNA sequencing libraries were constructed according to the manufacturers’ instructions.DNA sequencing was performed by the Scientific Operations core at the WSI on Pacific Biosciences SEQUEL II (HiFi) and Illumina NovaSeq 6000 (10X) instruments. Hi-C data were also generated from head and thorax tissue of iyOphLute1 using the Arima2 kit and sequenced on the Illumina NovaSeq 6000 instrument.

### Genome assembly, curation and evaluation

Assembly was carried out with Hifiasm ( Cheng *et al.*, 2021) and haplotypic duplication was identified and removed with purge_dups ( Guan *et al.*, 2020). One round of polishing was performed by aligning 10X Genomics read data to the assembly with Long Ranger ALIGN, calling variants with FreeBayes ( Garrison & Marth, 2012). The assembly was then scaffolded with Hi-C data ( Rao *et al.*, 2014) using YaHS ( Zhou *et al.*, 2023). The assembly was checked for contamination and corrected as described previously ( Howe *et al.*, 2021). Manual curation was performed using HiGlass ( Kerpedjiev *et al.*, 2018) and Pretext ( Harry, 2022). The mitochondrial genome was assembled using MitoHiFi ( Uliano-Silva *et al.*, 2023), which runs MitoFinder ( Allio *et al.*, 2020) or MITOS ( Bernt *et al.*, 2013) and uses these annotations to select the final mitochondrial contig and to ensure the general quality of the sequence.

A Hi-C map for the final assembly was produced using bwa-mem2 ( Vasimuddin *et al.*, 2019) in the Cooler file format ( Abdennur & Mirny, 2020). To assess the assembly metrics, the *k*-mer completeness and QV consensus quality values were calculated in Merqury ( Rhie *et al.*, 2020). This work was done using Nextflow ( Di Tommaso *et al.*, 2017) DSL2 pipelines “sanger-tol/readmapping” ( Surana *et al.*, 2023a) and “sanger-tol/genomenote” ( Surana *et al.*, 2023b). The genome was analysed within the BlobToolKit environment ( Challis *et al.*, 2020) and BUSCO scores ( Manni *et al.*, 2021; Simão *et al.*, 2015) were calculated.

Table 3 contains a list of relevant software tool versions and sources.

**Table 3.** Software tools: versions and sources.

Software tool	Version	Source
BlobToolKit	4.0.7	https://github.com/blobtoolkit/blobtoolkit
BUSCO	5.3.2	https://gitlab.com/ezlab/busco
FreeBayes	1.3.1-17-gaa2ace8	https://github.com/freebayes/freebayes
gEVAL	N/A	https://geval.org.uk/
Hifiasm	0.15.3	https://github.com/chhylp123/hifiasm
HiGlass	1.11.6	https://github.com/higlass/higlass
Long Ranger ALIGN	2.2.2	https://support.10xgenomics.com/genome-exome/software/pipelines/latest/advanced/other-pipelines
Merqury	MerquryFK	https://github.com/thegenemyers/MERQURY.FK
MitoHiFi	2	https://github.com/marcelauliano/MitoHiFi
PretextView	0.2	https://github.com/wtsi-hpag/PretextView
purge_dups	1.2.3	https://github.com/dfguan/purge_dups
sanger-tol/genomenote	v1.0	https://github.com/sanger-tol/genomenote
sanger-tol/readmapping	1.1.0	https://github.com/sanger-tol/readmapping/tree/1.1.0
YaHS	1.0	https://github.com/c-zhou/yahs

### Genome annotation

The BRAKER2 pipeline ( Brůna *et al.*, 2021) was used in the default protein mode to generate annotation for the *Ophion slaviceki* assembly (GCA_944452715.1) in Ensembl Rapid Release.

### Wellcome Sanger Institute – Legal and Governance

 The materials that have contributed to this genome note have been supplied by a Darwin Tree of Life Partner. The submission of materials by a Darwin Tree of Life Partner is subject to the **‘Darwin Tree of Life Project Sampling Code of Practice’**, which can be found in full on the Darwin Tree of Life website here. By agreeing with and signing up to the Sampling Code of Practice, the Darwin Tree of Life Partner agrees they will meet the legal and ethical requirements and standards set out within this document in respect of all samples acquired for, and supplied to, the Darwin Tree of Life Project.

Further, the Wellcome Sanger Institute employs a process whereby due diligence is carried out proportionate to the nature of the materials themselves, and the circumstances under which they have been/are to be collected and provided for use. The purpose of this is to address and mitigate any potential legal and/or ethical implications of receipt and use of the materials as part of the research project, and to ensure that in doing so we align with best practice wherever possible. The overarching areas of consideration are:

- Ethical review of provenance and sourcing of the material
- Legality of collection, transfer and use (national and international)

Each transfer of samples is further undertaken according to a Research Collaboration Agreement or Material Transfer Agreement entered into by the Darwin Tree of Life Partner, Genome Research Limited (operating as the Wellcome Sanger Institute), and in some circumstances other Darwin Tree of Life collaborators.

## Data Availability

European Nucleotide Archive: *Ophion slaviceki.* Accession number PRJEB53249; https://identifiers.org/ena.embl/PRJEB53249. ( Wellcome Sanger Institute, 2022) The genome sequence is released openly for reuse. The *Ophion slaviceki* genome sequencing initiative is part of the Darwin Tree of Life (DToL) project. All raw sequence data and the assembly have been deposited in INSDC databases. Raw data and assembly accession identifiers are reported in Table 1.

## Author information

Members of the Natural History Museum Genome Acquisition Lab are listed here: https://doi.org/10.5281/zenodo.4790042.

Members of the Darwin Tree of Life Barcoding collective are listed here: https://doi.org/10.5281/zenodo.4893703.

Members of the Wellcome Sanger Institute Tree of Life programme are listed here: https://doi.org/10.5281/zenodo.4783585.

Members of Wellcome Sanger Institute Scientific Operations: DNA Pipelines collective are listed here: https://doi.org/10.5281/zenodo.4790455.

Members of the Tree of Life Core Informatics collective are listed here: https://doi.org/10.5281/zenodo.5013541.

Members of the Darwin Tree of Life Consortium are listed here: https://doi.org/10.5281/zenodo.4783558.
