# Supplementary material for: The genome sequence of an ichneumon wasp, Ophion slaviceki (Kriechbaumer, 1892)
Source: Wellcome Open Res. 2023 Sep 8;8:397. [Version 1] doi: 10.12688/wellcomeopenres.19996.1 (PMC11224712; doi:10.12688/wellcomeopenres.19996.1)
Supplement: Peer review history

# Reviewer report: Reviewer response for version 1

**DOI:** 10.21956/wellcomeopenres.22142.r86973

**Published:** 2024-07-04

**Authors:** Elisabeth A Herniou

**Affiliations:** 1 Institut de Recherche sur la Biologie de l'Insecte, UMR 7261, CNRS - University of Tours, Tours, France

This the report describing the complete genome sequence of the parasitoid wasp *Ophion slaviceki*. This species is part of a species complex which was recently revised. It parasitised important pest species from the lepidtoperan genus Agrotis. This chromosomal assembly prodives invaluable data for the comparative genomic investigation of Ichneumonid wasps.

Are sufficient details of methods and materials provided to allow replication by others?

Yes

Is the rationale for creating the dataset(s) clearly described?

Yes

Are the datasets clearly presented in a useable and accessible format?

Yes

Are the protocols appropriate and is the work technically sound?

Yes

Reviewer Expertise:

Comparative genomics of Insects and insect virology

I confirm that I have read this submission and believe that I have an appropriate level of expertise to confirm that it is of an acceptable scientific standard.

# Reviewer report: Reviewer response for version 1

**DOI:** 10.21956/wellcomeopenres.22142.r86974

**Published:** 2024-07-04

**Authors:** Željko Tomanović

**Affiliations:** 1 Serbia University of Belgrade, Belgrade, Serbia

The author presented the genome of a nocturnal ichneumon wasp, *Ophion slaviceki*, previously thought to be *Ophion luteus*. The article gave an excellent background with known biology and ecology of the sequenced specimen. Technically and methodologically the article contains sufficient explanation and looks very good. The article is very well written. I recommend the article for indexing in presented form.

Are sufficient details of methods and materials provided to allow replication by others?

Yes

Is the rationale for creating the dataset(s) clearly described?

Yes

Are the datasets clearly presented in a useable and accessible format?

Yes

Are the protocols appropriate and is the work technically sound?

Yes

Reviewer Expertise:

diversity, ecology, phylogeny parasitoid wasps

I confirm that I have read this submission and believe that I have an appropriate level of expertise to confirm that it is of an acceptable scientific standard.
